# Synthesis of Cu(OH)_2_ nanowires modified by Fe_3_O_4_@SiO_2_ nanocomposite via green and innovative method with antibacterial activity and investigation of magnetic behaviours

**DOI:** 10.1098/rsos.212025

**Published:** 2022-06-01

**Authors:** Ensiye Zare-Bakheir, Mohammad Reza Ahghari, Ali Maleki, Hossein Ghafuri

**Affiliations:** Catalysts and Organic Synthesis Research Laboratory, Department of Chemistry, Iran University of Science and Technology, Tehran 16846-13114, Iran

**Keywords:** antibacterial activities, magnetic core–shell particles, recycling, nanocomposite, nanoparticles (NPs), Cu(OH)_2_-decorated Fe_3_O_4_@SiO_2_ core–shell nanospheres (FSCNWs)

## Abstract

In this study, green synthesis of modified Cu(OH)_2_ nanowires by Fe_3_O_4_@SiO_2_ core–shell nanospheres was easily performed via chemical reduction. In other words, the direct coating of Cu(OH)_2_ on Fe_3_O_4_@SiO_2_ was successfully realized without the extra complicated procedures. Various concentrations of synthesized nanocomposites were tested on pathogenic and nosocomial bacteria. In this study, the structural information and characterization of Fe_3_O_4_@SiO_2_/Cu(OH)_2_ nanowires (FSCNWs) were obtained using FE-SEM, FT-IR, EDX and X-ray diffraction. This nanocomposite can effectively kill important infectious bacteria, including *Staphylococcus aureus*, *Escherichia coli*, *Staphylococcus saprophyticus*, *Pseudomonas aeruginosa* and *Klebsiella pneumoniae*. Studies have shown that FSCNW nanocomposites affect common antibiotic-resistant bacteria. This result confirms the function of FSCNW as an effective, beneficial and environmentally friendly antibacterial agent that can used in a wide range of applications in medicine. FSCNWs can be separated conveniently from bacteria-containing solutions using a magnet. Compared with nanocomposites based on other metals such as silver and gold, the use of FSCNWs in water treatment has been recommended because of the precursor of copper for its low price and less toxicity. In addition to its special properties such as mild reaction conditions, green synthesis methods, admissible magnetic properties, easy separation, high antibacterial activity and beneficial efficiency.

## 1. Introduction

In recent years, water pollution has become a global concern, and harmful microorganisms such as viruses, fungi and bacteria in drinking water or water sources can cause many diseases, putting human health at risk. *Staphylococcus aureus* and *Escherichia coli* are among the bacteria that cause diarrhoeal diseases in humans through contaminated water. Therefore, a safe drinking water supply is vital for human health [1,2]. Chlorine, ozone and chloramine are examples of traditional disinfectants that can effectively suppress microbial infections. However, in practical applications, these disinfectants cause may carcinogenic by products that are detrimental to human health [3,4].

Nanotechnology has led to safer, more cost-effective and more environmentally friendly methods than traditional chemical disinfectants for removing microbial contaminants worldwide [5]. One of the most pressing topics for scientists in the field of nanotechnology is the development of biocompatible antibacterial agents [6]. Antibacterial agents have been used in various fields such as water purification, medicine, textiles and food packaging. Efficient and beneficial antibacterial agents are being developed to prevent pathogenic bacterial damage and emergence of antibiotic-resistant strains [7].

*Klebsiella pneumoniae*, *Staphylococcus saprophytic* and *Pseudomonas aeruginosa* are samples of pathogenic bacteria, and successful eradication of these bacteria has emerged as a clinically vital issue [8–11].

*Klebsiella pneumoniae* is a Gram-negative pathogen found extensively in the skin, mouth and intestine. In addition, it is responsible for a wide variety of nosocomial infections in immunodeficient patients and respiratory, urinary and gastrointestinal tract infections in humans [9,12].

Gram-positive bacterium *Staphylococcus saprophytic* is the second most common cause of acute urinary tract infection [11].

*Pseudomonas aeruginosa* is a pernicious opportunistic pathogen and a Gram-negative bacillus [10,13]. Owing to the extraordinary resistance of *P. aeruginosa* to antibacterial medicines, especially β-lactam antibiotics, including cephalosporins and carbapenem, their eradication has become increasingly challenging [10,14,15]. Thus, growing concerns regarding to evolution of bacteria resistant to common antibiotics have led to the widespread use of nanostructured materials with antibacterial properties such as copper, silver, gold and oxidized titanium [16,17]. Recently, copper nanoparticles (NPs) have received increasing attention among metal NPs and metal oxides. Copper-based nanomaterials with excellent antibacterial properties are suitable alternatives to antibacterial materials because of their abundance and low cost rather than silver [18–24].

In this study, to inhibit oxidation or dissolution in an aqueous solution and retain the initial magnetic activity of Fe_3_O_4_ (core), Fe_3_O_4_@SiO_2_ nanocomposite microspheres were first prepared by coating the Fe_3_O_4_ surface with a thin layer of SiO_2_ [25–31]. The magnetic cores ensured quick separation [32].

Furthermore, Cu(OH)_2_ nanowire-decorated Fe_3_O_4_@SiO_2_ core–shell nanospheres (FSCNWs) with high-performance antibacterial properties were synthesized by efficient procedures. Through combining materials with potential attributes, a composite material with higher effectiveness was created than its constituents. Therefore, Cu(OH)_2_ NPs were placed on the surfaces of FSCNWs. Eventually, owing to the unique properties of each component, low-cost synthesis and excellent antimicrobial activity, this nanocomposite is appropriate for water treatment.

In addition, the presence of magnetic NPs allows a magnet to save the nanocomposite from the reaction media. Also, high chemical stability has made it possible to re-use nanocomposites with a stable function in removing bacterial contaminations from drinking water.

**Scheme 1.**
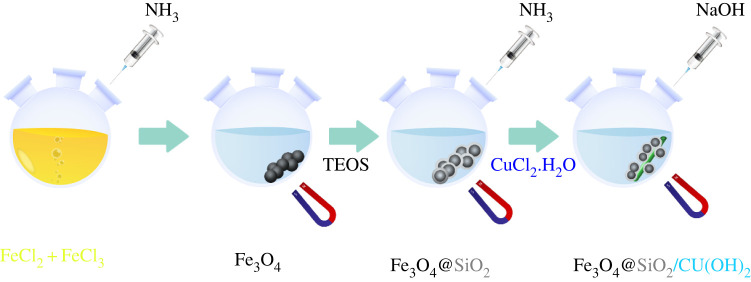
Preparation of Fe_3_O_4_@SiO_2_/Cu(OH)_2_.

Moreover, considering the effectiveness of the nanocomposite on antibiotic-resistant human pathogenic bacteria can be mentioned as a great advantage (scheme 1).

## 2. Material and methods

### 2.1. General

Pure materials such as solvents, metallic salts and chemical compounds were procured from Merck companies. The FE-SEM images were taken by using a Hitachi S-4160 microscope. The FT-IR spectrum was acquired using a Shimadzu IR-470 spectrometer with a KBr pellet. X-ray diffraction (XRD) was performed by using a D8 Advance model made via Bruker. Numerix DXP-X10P reported the EDX analysis of the nanocatalyst.

### 2.2. Preparation of Fe_3_O_4_@SiO_2_@Cu(OH)_2_

First, FeCl_3_.6H_2_O (20 mmol) and FeCl_2_.4H_2_O (20 mmol) were dissolved in 200 ml of distilled water to synthesize Fe_3_O_4_ via co-deposition. The mixture was agitated in a closed vessel at 80°C for 2 h. Then, by adding fresh NH_3_.H_2_O ammonia (25%) solution to the resultant solution, iron oxide NPs were obtained at pH = 12. Finally, the dark magnetic precipitate of Fe_3_O_4_ was produced and amassed through an external magnetic field and dried after three rinses with distilled water.

Second, 45 mg magnetic NPs were dispersed in water (16 ml) by ultrasonic waves for 15 min and mixed in fresh NH_3_.H_2_O (25 wt%) (2 ml) then ethanol (80 ml). In the next step, tetraethoxysilane (TEOS) (0.8 ml) was added to the system, and the pre-hydrolysis of TEOS lasted for 24 h under stirring. Then Fe_3_O_4_@SiO_2_ was collected at the bottom of the container using a magnet and dried at 70°C after rinsing with distilled water.

Finally, 0.1 g Fe_3_O_4_@SiO_2_ was added to 10 ml deionized water, the mixture was ultrasonicated for 15 min, and eventually 0.85 g CuCl_2_.H_2_O was added to reaction mixture. The mixture then adjusted to pH = 13 using 1.0 mol.l^−1^ NaOH aqueous solution so that the colour of the reaction solution changed to dark green at the end of the reaction [33–37].

### 2.3. General procedure for the antibacterial test

Before each microbiological test, samples and glassware were sterilized by autoclaving at 121°C for 15 min. Gram-negative and Gram-positive microorganisms such as *E. coli* and *S. aureus* were used for the antibacterial experiment. For this purpose, Mueller–Hinton agar containing plates, as a microorganism growth medium, with McFarland turbidity as a standard of 0.5, and the inhibition zone method were used for colony counting tests. For that, 3.8 g of Muller Hinton Agar powder was dissolved in 100 ml of distilled water and autoclave sterilized. The pH of the medium was generally maintained around the physiological pH of 7.4. Then, cooled agar solution (approximately 20 ml) was poured aseptically into each sterilized Petri dish. Finally, bacteria impregnated with a glass hockey stick were cultured on a solid agar medium in all directions and incubated at 37°C for 24 h. In addition, 0.5 McFarland turbidity standard, DMSO (0.1 ml) and NPs (0.1 g) were added to the nutrient broth culture medium. After incubation of the mixtures at 37°C, 0.1 ml of this mixture was preserved in an agar medium for the colony counting experiment [38,39].

## 3. Results and discussion

Fe_3_O_4_@SiO_2_/Cu(OH)_2_ was synthesized via a simple method with water as an eco-friendly and green solvent in the present investigative study. The synthesized FSCNWs were characterized using FE-SEM, EDX, XRD and vibrating sample magnetometer (VSM). Furthermore, antibacterial activities were examined using an agar disc diffusion and colony counter method.

### 3.1. Characterization of Fe_3_O_4_@SiO_2_/Cu(OH)_2_

**Figure 1.**
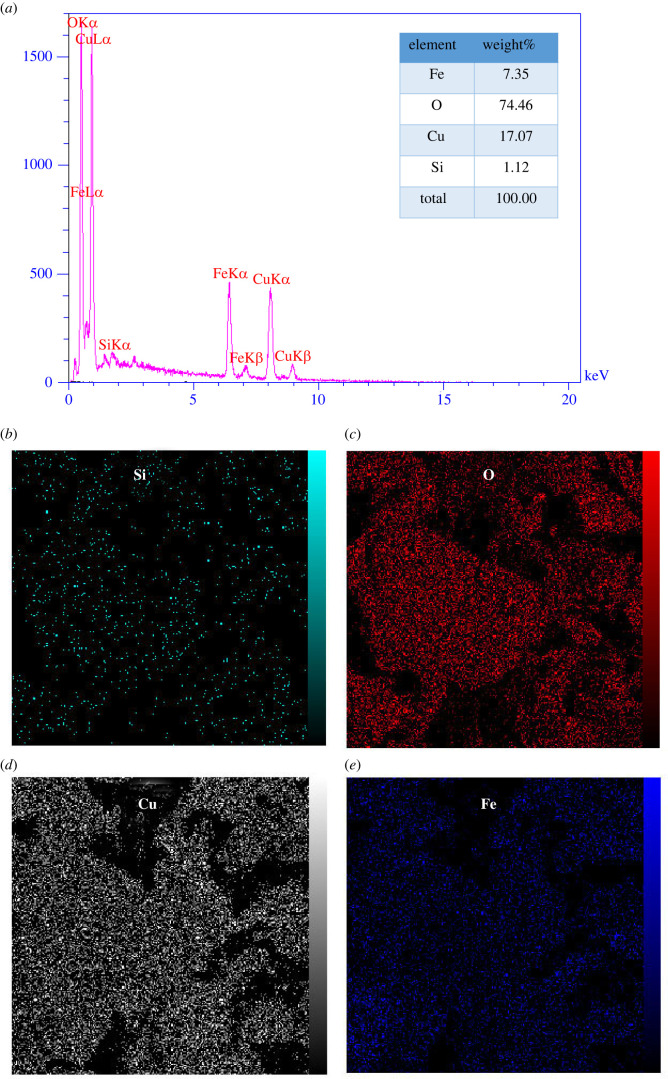
EDX spectrum (*a*) and mapping analysis of Fe_3_O_4_@SiO_2_/Cu(OH)_2_ (*b*) Si, (*c*) O, (*d*) Cu and (*e*) Fe.

The EDX analysis was applied as a typical technique to ensure the presence of copper, iron, silicon and oxygen in the synthesis Fe_3_O_4_@SiO_2_/Cu(OH)_2_, as presented in figure 1.

#### 3.1.1. Microscopic imaging study

**Figure 2.**
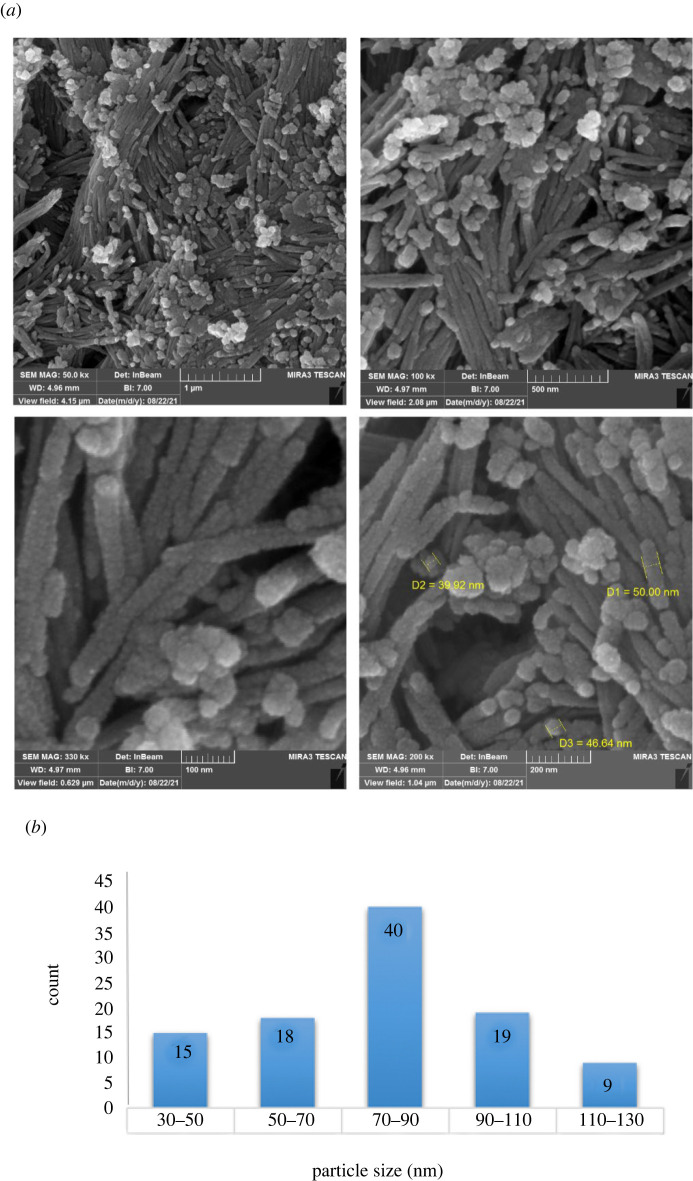
(*a*) FE-SEM images and (*b*) the particle size distribution curve.

The FE-SEM spectrum was used to investigate the morphology of the synthesized Fe_3_O_4_@SiO_2_/Cu(OH)_2_ and its highly uniform distribution (figure 2*a*). This image confirms the synthesis of Cu(OH)_2_ nanowires modified by Fe_3_O_4_@SiO_2_. The surface morphology of nanowires was observed with an average length and diameter of 1 µm and 55 nm, respectively. The FE-SEM images show that the aggregation of the NWs led to the formation of a wicker texture structure. Moreover, the results obtained from the particle size distribution diagram (figure 2*b*) showed that a large percentage of the nanowire diameter was smaller than 78 nm.

**Figure 3.**
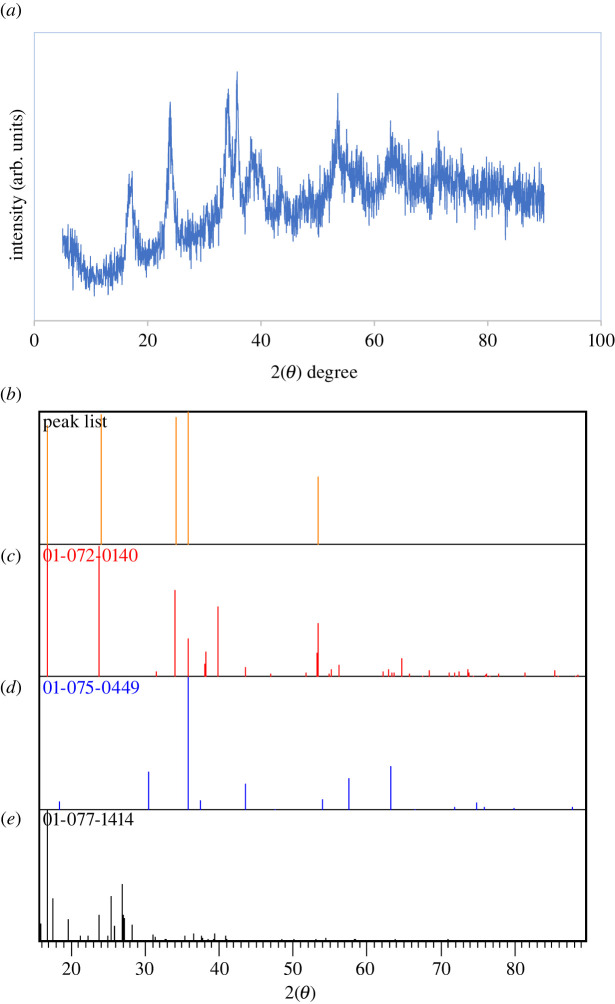
The XRD pattern of (*a*) Fe_3_O_4_@SiO_2_/Cu(OH)_2_, (*b*) the peak list of Fe_3_O_4_@SiO_2_/Cu(OH)_2_, (*c*) the reference of Cu(OH)_2_ (*d*) the reference of Fe_3_O_4_ and (*e*) the reference of SiO_2_.

The crystal structure of Fe_3_O_4_@SiO_2_/Cu(OH)_2_ was examined by XRD analysis (figure 3). The XRD patterns of FSCNWs synthesis showed the diffraction angle of 16.76°, 24.01°, 34.13°, 35.80° and 53.41° (figure 3*a*). Also, figure 3*b* is observed in the peak list of FSCNWs, and figure 3*c* is the Cu(OH)_2_ orthorhombic structure reference (JCPDS card no. 01-072-0140).

Figure 3*d* is Fe_3_O_4_ cubic structure reference (JCPDS card no. 01-075-0449), and figure 3*e* is SiO_2_ hexagonal structure reference (JCPDS card no. 01-077-1414), and patterns were quite consistent with the characteristic data of FSCNWs. Based on this information, the average crystallite size of FSCNWs was approximately 30 nm. Their crystallite size was computed by Debye–Scherrer using equation (3.1) as follows:

3.1 $$D_{P}=0.94\lambda /\beta \cos\theta$$

According to the above equation, the average crystal size (*D*_P_) is given by the X-ray wavelength (*λ*), the peak width at half the maximum height on the horizontal axis of the diffraction pattern (*β*) and the Bragg angle size (*θ*) [40–44].

#### 3.1.2. Vibrating sample magnetometer analysis

The VSM method evaluated the magnetic behaviour of the Fe_3_O_4_, Fe_3_O_4_@SiO_2_ and Fe_3_O_4_@SiO_2_/Cu(OH)_2_ nanocomposite at room temperature. Consequently, the magnetization (Ms) of Fe_3_O_4_ (56.5 emu g^−1^) and Fe_3_O_4_@SiO_2_ NPs (46.4 emu g^−1^) was higher than the Fe_3_O_4_@SiO_2_/Cu(OH)_2_ nanocomposite (7.2 emu g^−1^); also, data regarding remanence (Mr) and coercive force (Hc) are shown in table 1. As a result, the efficient linking of the Cu(OH)_2_ to the Fe_3_O_4_@SiO_2_ NPs is established.

**Table 1.** Magnetic parameters of Fe_3_O_4_ and Fe_3_O_4_@SiO_2_/Cu(OH)_2_.

sample	Ms (emu g^−1^)	Mr (emu g^−1^)	Hc (Oe)
Fe_3_O_4_	56.5	2.1	27.7
Fe_3_O_4_@SiO_2_	46.4	3.35	44.03
Fe_3_O_4_@SiO_2_/Cu(OH)_2_	7.2	0.8	52

**Figure 4.**
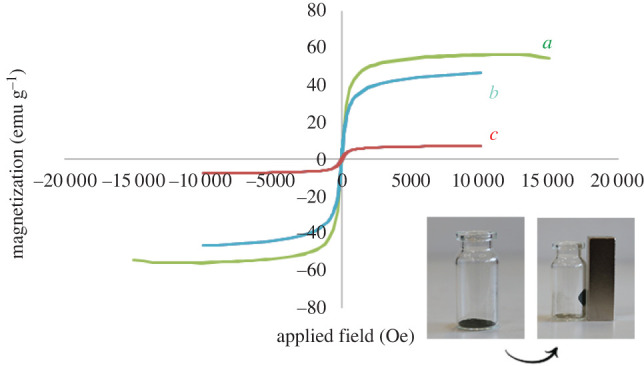
The room temperature M–H curve of (*a*) Fe_3_O_4_, (*b*) Fe_3_O_4_@SiO_2_ and (*c*) Fe_3_O_4_@SiO_2_/Cu(OH)_2_.

Heterogeneous paramagnetic nanocomposites are produced due to their significant advantages: unique surface properties, frequent use and the ability to simply segregate from the reaction mixture, which is essential due to the economical cost and time at an industrial scale (figure 4) [44,45].

#### 3.1.3. FT-IR spectrum of the Fe_3_O_4_@SiO_2_/Cu(OH)_2_ in the range of 400–4500 cm^−1^

**Figure 5.**
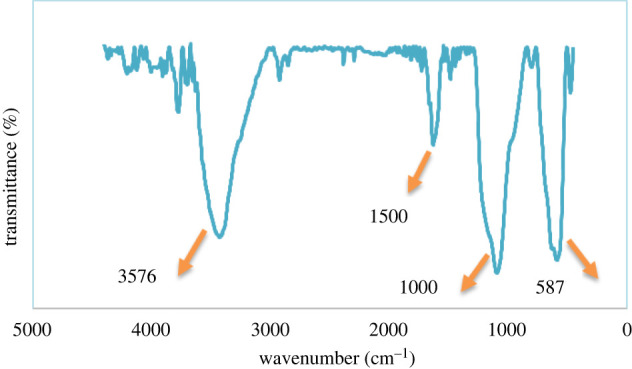
FT-IR spectra of Fe_3_O_4_@SiO_2_/Cu(OH)_2_.

FT-IR spectroscopy was carried out to investigate purity and the presence of compounds in the prepared FSCNWs. In the FT-IR spectra of Fe_3_O_4_@SiO_2,_ a wide peak has appeared at approximately 1090 cm^−1^ according to the O-Si-O stretching vibration, which confirms the favoured synthesis of Fe_3_O_4_@SiO_2._ The features of peaks at 587, 1000 and 3576 cm^−1^ are related to the Cu-O stretching vibration and Cu-O-H, as well as O-H stretching vibration proving the successful synthesis of coated Cu(OH)_2_ with Fe_3_O_4_@SiO_2_. In addition, 1500 cm^−1^ belongs to H-O-H bending vibrations of H_2_O adsorbed onto the FSCNWs lattice (figure 5) [35,46,47].

### 3.2. Antibacterial properties of Fe_3_O_4_@SiO_2_/Cu(OH)_2_, Fe_3_O_4_@SiO_2_ and Fe_3_O_4_

**Figure 6.**
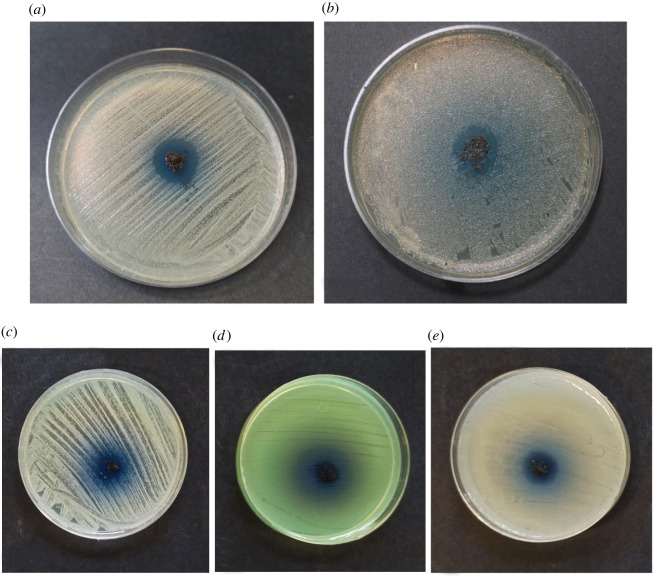
Images of Agar disc diffusions of (*a*) *S. aureus*, (*b*) *E. coli*, (*c*) *S. saprophyticus*, (*d*) *P. aeruginosa* and (*e*) *K. pneumoniae* in the presence of Fe_3_O_4_@SiO_2_/Cu(OH)_2_.

The antibacterial test of Fe_3_O_4_@SiO_2_@Cu(OH)_2_ was examined as an practical application. Agar disc diffusion as the most practical method was chosen to peruse the antibacterial activity of FSCNWs against *Staphylococcus aureus (S. aureus)* (ATCC 12600) and *Staphylococcus saprophyticus (S. saprophyticus)* (ATCC 1440), a Gram-positive bacterium, and *Escherichia coli (E. coli)* (ATCC 9637), *Klebsiella pneumoniae (K. pneumoniae)* (ATCC 700603) and *Pseudomonas aeruginosa (P. aeruginosa)* (ATCC 27853), a Gram-negative bacterium. As presented in figure 6, in the presence of 0.01 g FSCNWs at 37°C for 24 h, the diameters of the zones of inhibition (ZOI) were 0.5, 0.6, 0.5, 0.6 and 0.7 cm for *E. coli, S. aureus*, *S. saprophyticus*, *K. pneumoniae* and *P. aeruginosa,* respectively (figure 6).

The ZOIs around the circle-tested samples were measured to evaluate their comparative antibacterial efficiency against these five essential microorganisms.

For example, these variations in results may be attributed to the distinct bacterial cells. *Staphylococcus aureus* consists of a thick peptidoglycan layer sensitive to intracellular transmission, which causes cell wall fractures. By contrast, *E. coli* possesses a phospholipid bilayer cell membrane and a thin peptidoglycan layer [48–51].

**Figure 7.**
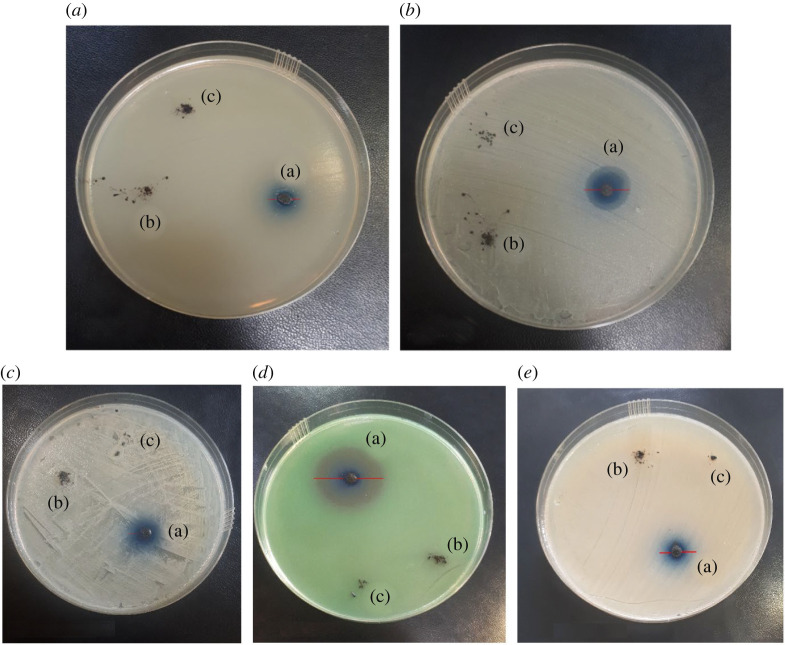
Images of Agar disc diffusions of (*a*) *E. coli*, (*b*) *S. aureus*, (*c*) *S. saprophyticus*, (*d*) *P. aeruginosa* and (*e*) *K. pneumoniae* in the presence of (a) Fe_3_O_4_@SiO_2_/Cu(OH)_2_, (b) Fe_3_O_4_@SiO_2_ and (c) Fe_3_O_4_ after 24 h.

In order to ensure the accuracy of the results, the antibacterial performances of Fe_3_O_4_ and Fe_3_O_4_@SiO_2_ nanomaterials rather than Fe_3_O_4_@SiO_2_/Cu(OH)_2_ nanocomposite were evaluated on several bacteria such as *S. aureus, E. coli*, *S. saprophyticus*, *P. aeruginosa* and *K. pneumoniae*. As shown in figure 7, Fe_3_O_4_ and Fe_3_O_4_@SiO_2_ components did not exhibit antibacterial properties. The antibacterial effect of the final composite was attributed to the antibacterial properties of Cu(OH)_2_ nanowires.

The colony counter method was used to assess the concentration of live bacteria in cultivated samples [52–56].

Aiming to evaluate, the antibacterial functions of increasing concentrations of Fe_3_O_4_@SiO_2_/Cu(OH)_2_ were compared with the results of the control obtained under identical conditions without Fe_3_O_4_@SiO_2_/Cu(OH)_2_.

**Figure 8.**
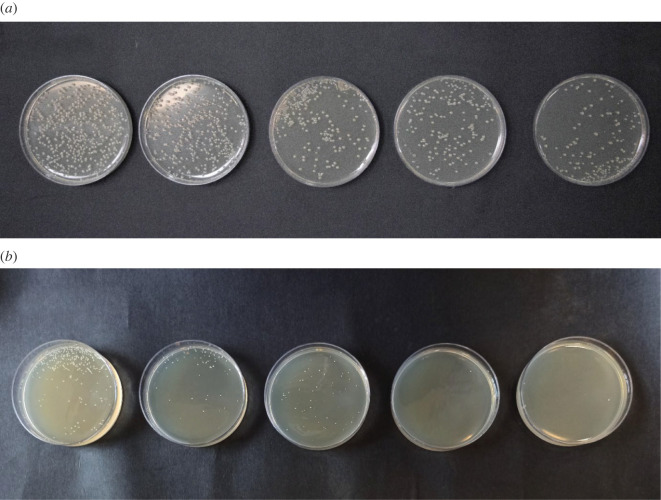
Photographs of (*a*) *E. coli* and (*b*) *S. aureus* in the absence and presence of Fe_3_O_4_@SiO_2_/Cu(OH)_2_ after 24 h.

According to the digital images shown in figure 8, FSCNWs inactivated *E. coli* and *S. aureus* pathogen bacteria after 24 h (figure 8 and table 2).

**Table 2.** Number of colony-forming units (CFU) of *E. coli* and *S. aureus* in the absence and presence of various concentrations Fe_3_O_4_@SiO_2_/Cu(OH)_2_ after 24 h.

Types	control	0.001 g ml^−1^	0.002 g ml^−1^	0.003 g ml^−1^	0.004 g ml^−1^
*E. coli* (Gram-negative)	236	146	120	96	61
*S. aureus* (Gram-positive)	312	90	70	7	2

### 3.3. Antimicrobial activity mechanism

The present study showed that Cu(OH)_2_ has antibacterial properties on *S. aureus* and *E. coli*, and the attendance of Cu(OH)_2_ has been linked to the production of reactive oxygen species (ROS).

When the particle size shrinks, the effects will be stronger and more pronounced. As a result, Cu(OH)_2_ nanostructures with a higher surface-to-volume ratio produced more ROS and were best at penetrating cell membranes. A bactericidal effect has been suggested due to the electrostatic interaction between NPs and the negatively charged bacterial cell membrane and the release of Cu^2+^ ions. Cu^2+^ may interact with DNA and proteins, causing structural changes and disrupting biological procedure [57–60].

Ksp is the equilibrium constant for extremely insoluble solids, computed by multiplying the molar concentration of anions and cations dissolved in the saturated aqueous solution, which is 2.2 × 10^−20^ for Cu(OH)_2_ by their respective solubility products [51].

## 4. Conclusion

In the present study, a new, practical, low-cost, re-usable, heterogeneous Fe_3_O_4_@SiO_2_/Cu(OH)_2_ nanocomposite was produced for the first time and designed by a green and effective method at low temperature for a short time. FSCNWs have been synthesized at low temperatures and atmospheric pressure without hazardous chemicals, indicating facile operation.

The results of the present study suggest that copper, rather than silver, could be used as an antiseptic in the processing of drinking water with lower risk to human health. Furthermore, FSCNWs are far less expensive than silver and superior for commercial uses either in economic or environmental aspects; thus, they are expected to be more widely used.

Furthermore, these Cu(OH)_2_ nanowires modified by Fe_3_O_4_@SiO_2_ magnetic nanocomposite as a highly useful re-usable nanomaterial was separated by a magnet for antibacterial activity.

In addition, the FSCNWs revealed excellent antibacterial properties. For this purpose, the antibacterial properties of Fe_3_O_4_@SiO_2_/Cu(OH)_2_ against *E. coli* and *S. aureus* were tested using agar disc diffusion and colony counter methods. Nevertheless, *S. aureus* demonstrated a higher ZOI than *E. coli* bacterium. Moreover, the colony method confirmed the inactivation of *S. aureus* and *E. coli* bacteria in the presence of FSCNWs. The results of the present study have suggested that these nanocomposites as cost-effective antibacterial agents could be very helpful in water purification and industrial applications.

Also, bacteria such as *S. saprophytic*, *P. aeruginosa* and *K. pneumoniae* cause serious diseases in humans. Therefore, the effect of FSCNWs on certain antibiotic-resistant and human pathogenic bacteria makes them a suitable option for biotechnology and therapeutic applications. Finally, the FSCNWs were effective against five pathogenic bacterial strains.

In addition, the VSM analysis and easy separation corroborated its magnetic properties, and the obtained EDX, XRD and IR results agreed with the proposed structure; moreover, these magnetic nanocomposites with nanowire structures and diameters average of 75 nm were fully characterized through FE-SEM.

## Data Availability

The datasets supporting this article have been uploaded as part of the electronic supplementary material [61]. Images of repeat colony test on *E. coli* and *S. aureus* in the presence and absence of Fe_3_O_4_@SiO_2_@Cu(OH)_2_ after 24 h are in the electronic supplementary information section. Also, the images resulting from the inhibition zone method in the presence of Fe_3_O_4_ and Fe_3_O_4_@SiO_2_ after 24 h are available in the article version on the publisher's website.
